# Everybody's Page

**Published:** 1891-08-15

**Authors:** 


### Everybody's Page.

It is rather hard upon the exponents of the "faith cure" to say that the difference between it and the "mind cure" is that the latter does not require any faith, and the "faith cure" does not require any mind.

Tobacco is not a necessary for the youth of England. So declares his honour Judge Bayley, who, by his decision in a case recently brought before him, has earned the gratitude of the stern British matron. It certainly sounds rather a startling assertion that tobacco is necessary for an infant! Be not frightened, fond mothers, we are writing of legal infants of less than twenty-one summers, not of babies in arms.

A curious instance of sudden death is reported from Washington. An inmate of the workhouse, after eating a hearty dinner—we notice that inmates of workhouses invariably eat hearty meals—rose from the table, and after walking a few steps to the door fell down and almost immediately died. At the autopsy which followed, the brain, heart, lungs, viscera, etc., were examined without showing anything to account for the sudden collapse. But on opening the larynx the cause was revealed. A piece of gristly corned beef, one inch long and half an inch thick was firmly wedged in the throat. Had the man followed the precept of a well known politician, and given thirty-two bites to each mouthful, he probably would have lived to enjoy many more hearty meals.

Some sensible remarks on the subject of "taking cold" and its frequently disastrous consequences, together with advice on the subject of clothing, are to be found in a little book entitled "Taking Cold," by Dr. F. H. Bosworth, published in Detroit. Though everyone will not be inclined to agree with all his maxims, there is plenty of instruction to be gleaned from his pages. His attacks on some absurdities of dress occasionally carry him too far. In his anxiety to get rid of the cumbersome headgear of civilization he omits to substitute anything in the way of protection from the sun. The only recommendations of silk in his eyes are fashion and its high price, but he is near the mark when he suggests that many people think they have done all that is necessary to secure warmth by wearing heavy clothing irrespective of its material and capacity for imparting warmth.

Elephants are not such strong animals as we thought them to be. It only takes four men to administer a dose of castor oil to one of these creatures, and it sometimes takes as many, if not more, people to administer the drug to a child.

Even in these days of surprises it is too much to expect such a novelty as the deviation into grammatical accuracy of an Act of Parliament. But it is a subject for congratulation to find that our legislators can occasionally be practical in their paternal efforts to look after our moral behaviour. With a view to nip in the bud effectually the growing form of inebriety caused by the drinking of methylated spirit in the North of Ireland, the Government has caused the spirit to be made much more nauseous.

So long as the British workman is content to live for the day it seems hopeless to protest against the unrestricted influx of destitute aliens into this country. Coupled with the fact that there is annually a large emigration of some of our ablest-bodied citizens, and that the recent census does not show the increase in population that was looked for, the addition of foreign refuse to our own dregs does not promise well for the future prosperity of our labouring classes. They complain of foreign competition; it is in their power to lessen it even though they cannot abolish it. They are much more practical in these matters in America.

Is it not going into extremes to lay down as a hard and fast rule that no two persons can habitually sleep together without loss of health, that *invariably* one will thrive, and the other lose? Yet it is a curious fact, that if a young child sleeps in the same bed with an elderly person the child does not thrive, and no doubt it would be better if the custom of separate beds were more universal. According to a French authority much of the nervousness and discomfort which people complain of when they rise in the morning is due to the fact that each does not sleep alone, and that there are electrical changes going on in the system during the night which work destructive results to those who sleep together night after night under the same bedding. Whether this electrical bogie is sneaking about under the bedding or in the brain of this authority we do not know.

